# Development of a national dataset for geospatial analysis of congenital heart disease

**DOI:** 10.3389/fped.2022.952048

**Published:** 2022-08-10

**Authors:** Jennifer H. Klein, Anand Gourishankar, Anita Krishnan

**Affiliations:** ^1^Division of Cardiology, Children's National Hospital, Washington, DC, United States; ^2^Division of Hospital Medicine, Children's National Hospital, Washington, DC, United States

**Keywords:** geospatial analysis, multicenter, geomapping, congenital heart disease, clustering

## Introduction

The purpose of this opinion commentary is to describe a novel approach for studying the geospatial distribution of congenital heart disease. Congenital heart disease is estimated to occur in 1 in 100 children born in the United States each year (1, 2). The geographic location of congenital heart disease cases has emerged as an area of interest, especially as there is increased focus on health equity, delivery of care, and social determinants of health. However, developing a better understanding of the geographic characteristics and its impact on health (or disease, e.g., congenital heart disease) presents formidable challenges. We describe opportunities and challenges in developing a national dataset for multicenter geospatial analysis in congenital heart disease.

## Context of geospatial analysis

Geospatial analysis, including the creation of geospatial maps—known colloquially as geomapping—is used across diverse industries, ranging from environmental conservation and national security to product delivery. It has a variety of public health applications including disease surveillance, emergency preparedness and response, community health, and environmental health (3). In healthcare, geospatial analysis aids in the study of disease etiology, influences, and outcomes, and can provide visualization tools to reinforce a conclusion, such as geographic disparities. For example, geospatial analysis tools can study relatedness of cases for spatial clustering, report drive time to tertiary care centers, analyze distance to a nearest point (such as a surgical center), or evaluate for association with an area's given attribute (percentage of high school graduates, or concentration of environmental air pollutants, for instance) (3, 4). Geographic Information System (GIS) with appropriate resources (e.g., software, hardware, and networking) and spatial analysis methods, including modeling (spatial, temporal, or both), provide a better understanding of the spatial organization of health care (5). Geography can thus provide a lens for understanding the cultural, socioeconomic, and built environment.

## Application in congenital heart disease

Pediatric cardiology has embraced the study of geographic location as a key factor in caring for patients. The Baltimore-Washington Infant Study Group, a seminal population health study in cardiology, described variations in congenital heart malformations by race and socioeconomic status (6). Since that time, a compelling body of evidence highlighting significant sociodemographic disparities in many aspects of congenital heart disease care has evolved. Within single centers or regions, there is demonstrated geographic variation of the incidence of congenital heart disease (7, 8). Several other studies have evaluated sociodemographic factors' impact on surgical mortality (9, 10) and long-term outcomes from congenital heart disease (11–14). Geographic location and other sociodemographic factors are also implicated in missed prenatal detection of congenital heart disease (15–17).

Applying geospatial analysis as a method to study geographic and sociodemographic factors is a novel but powerful approach that has yet to be fully explored in pediatric cardiology. Researchers have published a single-center experience on geospatial analysis of specific cardiac diagnoses, such as Tetralogy of Fallot, hypoplastic left syndrome, and d-transposition of the great arteries (8, 18). However, to the best of our knowledge, no studies have carried out geospatial analysis of congenital heart disease on a multicenter level. The prior studies have been within a single defined region, examining cases in a single referral center, state, or catchment area. In our opinion, multicenter geospatial analysis allows for a broader investigation of risk factors for congenital heart disease, associations with the social disadvantage that may impact care, and predict where care is needed. A multicenter approach thus allows for new investigations that would otherwise be impossible.

## Methods and framework for multicenter geospatial analysis

The authors seek to establish a national dataset to study prenatal detection of congenital heart disease. Through collaboration with the Fetal Heart Society, a multicenter research collaborative, partner sites are recruited for data sharing. Collaboration across several departments within Children's National Hospital provides the foundation for this endeavor, requiring input from pediatric cardiologists, epidemiologists, legal experts, and statisticians. In addition, the Child Health Advocacy Institute at Children's National Hospital provides geospatial expertise and technical support. The guiding principles of this approach are as follows:

1. *Partners:* Not only does a national dataset require research partners across the country, but also a team of geospatial analysts, epidemiologists, or statisticians familiar with geospatial analysis. This team approach is key to building a research network and performing rigorous geospatial analyses.
2. *Privacy:* Patient protection and privacy must be at the forefront of all data-sharing agreements. In addition to rigorously subscribing to Health Insurance Portability and Accountability Act and Institutional Review Board regulations, certain analyses may be precluded or might require redaction to protect patient privacy.
3. *Products:* Geographic information systems and geospatial analysis require advanced software or tools to perform the analyses and create maps. Several such tools exist. The authors have used ArcGIS^®^ from Esri (Redlands, CA) and continue to investigate new or alternative tools with varying functionalities and strengths.

## Discussion of challenges to multicenter geospatial analysis

Developing a national dataset of congenital heart disease poses noteworthy challenges. First, the selection and recruitment of partner centers require an investment of time, interest, and resources from each site. As partner sites are selected, it is also important to accurately represent the population desired to be sampled. Too few sites, or sites only within particular geographic regions or with specific demographic characteristics, may skew the analyses. Second, ensuring the security of protected health information may hinder the collection of data, as partner sites are appropriately cautious when sharing protected health information and may limit the types of analyses that can be performed. Geospatial analysis is most accurate when using the most specific level of detail available, meaning that street address is preferred to census tract, which is preferred to counties. However, as street address is considered protected health information, there may be limits to obtaining and analyzing addresses from multiple centers. Nevertheless, geographic manipulation (e.g., aggregation, masking, cross-hatching) and tabular methods (e.g., cell suppression, omission) can mitigate these privacy issues.

Third, planning suitable analyses presents challenges. First, spatial analysis is affected by lack of continuity in a given study area. For example, hot spot analysis is only feasible in a contiguous region. Hot spot analysis looks for non-random distribution of points in the form of statistically high or low clusters, such as was described in the case of environmental biodiversity (19). Extent of clustering is influenced by the area over which the analysis is performed (20). With partner centers contributing data from geographically and demographically unique settings, finding a common denominator by which settings can be compared poses a challenge. Furthermore, using multicenter datasets to perform clustering analyses—a particularly useful form of geospatial analysis—can be ripe for error, highlighting problems with inconsistent boundaries (20, 21), such as with varying hospital catchment areas. In addition to the geographic challenges to data analysis, there are also statistical challenges, particularly as pediatric cardiology studies relatively rare diseases. The small number problem with numerator (cases) or denominator (population or live births) creates rate instability and imprecise estimates. In summary, identifying the most appropriate analyses is dependent on the questions posed, the data available, and the needs of the population served.

Potential solutions to each of the three challenges are outlined in Table 1.

**Table 1:** Challenges and solutions to multicenter geospatial analysis.

**Challenge**	**Potential solutions**
Recruitment of partner centers for data sharing	➢ Use of existing networks/consortiums and prior research collaborators to recruit partner centers
Legal agreements for data sharing	➢ Data-coordinating center for streamlined data sharing
	➢ Consider options for sharing of de-identified patient data or non-protected health information geographic data
	➢ Use secure cloud service or data management software when cloud-based GIS solutions are unavailable across multiple centers
Planning appropriate multicenter analyses	➢ Tailor analyses to questions posed and data available
	➢ Engage geospatial analysis experts
	➢ Use geospatial techniques (such as spatial smoothing, collapsing, or combining data), as relevant to research question

## Conclusion

In this opinion we present the initial experience in creating a dataset of infants with congenital heart disease for the purpose of geospatial analysis. The fundamental principles of partnership, privacy, and products will enable success. The knowledge obtained from a single-center geospatial study is invaluable, but the lessons from a multicenter approach could be transformative for understanding congenital heart disease nationwide. In summary, we recommend the use of geospatial analysis to further understand disparities in incidence and outcomes within pediatric cardiology.

## Author contributions

JK conceived of and drafted this opinion piece. AG and AK provided content expertise and manuscript revisions. All authors contributed to the article and approved the submitted version.

## Funding

This work was supported by the American Heart Association Grant #821139/JK/2021.

## Conflict of interest

The authors declare that the research was conducted in the absence of any commercial or financial relationships that could be construed as a potential conflict of interest.

## Publisher's note

All claims expressed in this article are solely those of the authors and do not necessarily represent those of their affiliated organizations, or those of the publisher, the editors and the reviewers. Any product that may be evaluated in this article, or claim that may be made by its manufacturer, is not guaranteed or endorsed by the publisher.
